# Comparison of the diagnostic ability of Moorfield’s regression analysis and glaucoma probability score using Heidelberg retinal tomograph III in eyes with primary open angle glaucoma

**DOI:** 10.4103/0301-4738.71681

**Published:** 2010

**Authors:** Shveta Jindal, Tanuj Dada, V Sreenivas, Viney Gupta, Ramanjit Sihota, Anita Panda

**Affiliations:** Department of Ophthalmology, Dr. Rajendra Prasad Center for Ophthalmic Sciences, India; 1 Department of Biostatistics, All India Institute of Medical Sciences, New Delhi, India

**Keywords:** Confocal scanning laser ophthalmoscopy, glaucoma probability score, Heidelberg retinal tomograph

## Abstract

**Purpose:**

To compare the diagnostic performance of the Heidelberg retinal tomograph (HRT) glaucoma probability score (GPS) with that of Moorfield’s regression analysis (MRA).

**Materials and Methods:**

The study included 50 eyes of normal subjects and 50 eyes of subjects with early-to-moderate primary open angle glaucoma. Images were obtained by using HRT version 3.0.

**Results:**

The agreement coefficient (weighted k) for the overall MRA and GPS classification was 0.216 (95% CI: 0.119 – 0.315). The sensitivity and specificity were evaluated using the most specific (borderline results included as test negatives) and least specific criteria (borderline results included as test positives). The MRA sensitivity and specificity were 30.61 and 98% (most specific) and 57.14 and 98% (least specific). The GPS sensitivity and specificity were 81.63 and 73.47% (most specific) and 95.92 and 34.69% (least specific). The MRA gave a higher positive likelihood ratio (28.57 vs. 3.08) and the GPS gave a higher negative likelihood ratio (0.25 vs. 0.44).The sensitivity increased with increasing disc size for both MRA and GPS.

**Conclusions:**

There was a poor agreement between the overall MRA and GPS classifications. GPS tended to have higher sensitivities, lower specificities, and lower likelihood ratios than the MRA. The disc size should be taken into consideration when interpreting the results of HRT, as both the GPS and MRA showed decreased sensitivity for smaller discs and the GPS showed decreased specificity for larger discs.

Confocal scanning laser ophthalmoscopy by Heidelberg retinal tomography (HRT) has become a common test in all tertiary centers for detection of glaucoma and its progressive effect on the optic nerve head (ONH). It provides an objective and reproducible analysis of the optic disc morphological parameters.[1] One algorithm of the HRT is the Moorfields regression analysis (MRA), which has been developed to improve the diagnostic ability of HRT by considering differences in the area of the optic disc in the quantitative evaluation of the rim area.[2] This measure is limited by the need for an examiner to approximate the optic disc margin with a contour line to calculate the stereometric parameters and the MRA. This requirement added an undesirable subjectivity to the examination and may have resulted in significant differences in the topographic parameter values obtained by different examiners.[3]

A recently released version, advanced glaucoma analysis 3.0 (HRTIII), is an enhanced version of the previous HRTII software. The HRTIII provides the well-known MRA algorithm and a new one, the glaucoma probability score (GPS), which does not rely on a manually drawn contour line. GPS analyzes the shape of the patient’s anatomical optic ONH structure, independent of the contour line, using a 3-D model of optic disc and peripapillary retinal nerve fiber layer (RNFL).[4] It calculates a probability of structural abnormality, based on how closely the patient’s model compares to healthy and glaucomatous shapes.

This study compares the diagnostic performance of MRA and GPS in eyes, with early-to-moderate primary open angle glaucoma.

## Materials and Methods

A prospective cross-sectional case control study was conducted on one eye of 50 normal, healthy subjects and 50 subjects with early-to-moderate primary open angle glaucoma. In subjects where both the eyes were eligible for the study, the study eye was selected randomly by a set of computer generated random numbers. All subjects were ≥ 35 years, with a refractive error within ± 5 diopter (D), astigmatism within ± 3 D, and a best corrected visual acuity of ≥ 20/40. Subjects with significant media opacity (corneal, lenticular), any other intraocular or neurological diseases affecting the RNFL, optic disc, or visual field were excluded from the study.

The study was approved by the institutional ethics committee board. An informed consent was taken from all the participants. The subjects underwent a comprehensive ocular examination with a detailed medical history, slit lamp biomicroscopy, applanation tonometry, gonioscopy, stereoscopic dilated fundus examination, and visual field charting on Humphrey’s field analyzer 30-2 SITA (Swedish interactive threshold algorithm) Standard. Two clinically defined groups were included: normal healthy subjects and glaucomatous subjects. The normal subjects had no family history of glaucoma, an intraocular pressure (IOP) of ≤ 21 mmHg, open angles on gonioscopy, normal clinical evaluation, and a normal Humphrey visual field 30-2 SITA Standard. Glaucomatous eyes had an IOP of > 21 mm Hg at diagnosis, open angles on gonioscopy, glaucomatous optic nerve head changes, and visual field changes, typical of glaucoma (three contiguous non-edge points depressed with *P* < 5% probability of being normal, one of which had *P* < 1%, all being not contiguous with the blind spot, with glaucoma hemifield test outside normal limits, confirmed on two consecutive tests, pattern standard deviation (PSD) < 5% confirmed on two consecutive visual fields).[5]

Glaucomatous visual field defects were classified into early (mean deviation(MD) > - 6 db, fewer than 18 points depressed below 5% probability level and fewer than 10 points below the *P* < 1% level, no point with sensitivity of < 15 db in the central 5 degrees of fixation, and moderate (MD - 6 db to - 12 db, fewer than 37 points depressed below 5% probability level and fewer than 20 points below the *P* < 1% level, no absolute deficit (0 dB) in the 5 central degrees, and only one hemifield with sensitivity of < 15 dB in the central 5 degrees of fixation). Eyes with advanced visual field defects were excluded from the study.

The HRT is an ophthalmoscope that uses a confocal scanning diode laser with a wavelength of 670 nm, which can scan the retinal surface at multiple consecutive parallel focal planes. All HRT III images obtained were good quality, defined as having a topographic standard deviation of less than 30 μm and no floaters or opaque areas. Magnification errors were corrected by using patients’ corneal curvature measurements.

For the MRA, the ONH contour line is drawn with the ONH margin defined as the inner border of the Elschnig’s ring. In our study, the contour line was drawn by a single operator. The MRA compares the neuroretinal rim area globally and individually in six sectors with values predicted for a healthy subject with the same disc size and age. The result is recorded as a categorical classification: outside normal limits (ONL), borderline (BL), and within normal limits (WNL), depending on whether the observed rim area is smaller than 95% predicted limits (classified as BL) or smaller than 99.9% predicted limits (classified as ONL).

The GPS is obtained using a new automated analysis, independent of the contour line tracing. The software uses two measures of the peripapillary nerve fiber layer (horizontal and vertical RNFL curvature) and three measures of the ONH shape (cup depth, rim steepness, cup size) as an input into the relevance vector machine learning classifier. This provides a numerical index ranging from 0 (low probability of disease) to 1 (high probability of disease), to describe the estimated probability of finding similar data in the glaucoma group of the training data. Accordingly, the GPS output is then automatically classified into three categories: ONL; (GPS > 0.64), BL; (GPS between 0.24 and 0.64) and WNL; (GPS < 0.24).

All statistical analysis was calculated using the SPSS software version 15.0 and the STATA version 9.1 statistical software. Differences among groups were assessed by a Student’s *t* test for continuous parameters and Chi-square test for categorical parameters. The receiver operating characteristic (ROC) curves were used to assess the usefulness of each parameter, and sectors for differentiating glaucomatous eyes from healthy eyes. Sensitivities at 95% (5% false positive rate) fixed specificity were calculated for all global stereometric parameters. The diagnostic abilities of the color coded MRA results and color coded GPS classifications were calculated. Positive and negative likelihood ratios were calculated for these classifications. *k* statistics were used to analyze the agreement between MRA and GPS classifications.

## Results

Fifty eyes of 50 normal subjects and 50 eyes of subjects with early-to-moderate glaucoma were included. The baseline demographic and clinical characteristics are shown in Table 1. The patients with glaucoma were older (mean age difference, 14 years; *P* < 0.001) than the healthy subjects. The mean axial length for the normal subjects was 22.94 ± 0.71 mm and that for the glaucoma group was 23.5 + 0.91 mm (*P* = 0.001). The mean refractive error for the normal subjects was + 0.41(± 0.97) D and that for the glaucoma group was - 0.23 (± 1.95) D (*P* = 0.04). The average visual field MD for the healthy population was - 2.82 ± 1.36 db and for the glaucoma group was - 6.45 ± 2.97 db reflecting an early-to-moderate degree of glaucomatous damage.

**Table 1:** Baseline characteristics of the study population

Parameter	Normal (n = 50)		Glaucoma (n = 50)		Significance *P* value
	Mean	SD	Mean	SD	
Age (years)	44.74	8.88	58.78	11.08	*P* < 0.001
CCT* (mm)	537	27.77	530.8	27.99	*P* = 0.27
Mean deviation (db)	- 2.82	1.36	- 6.45	2.97	*P* < 0.001
Pattern standard deviation (db)	2.50	1.19	5.71	3.23	*P* < 0.001
Cup-disc ratio	0.36	0.11	0.71	0.13	*P* < 0.001

* CCT: Central corneal thickness

Overall, the GPS gave higher false positive results (28%) and the MRA gave higher false negative results (42%). Sensitivities and specificities were evaluated for the overall MRA and GPS classifications by using the most specific criteria (considering BL cases as test negatives) and the least specific criteria (considering BL cases as test positives). Overall, the GPS had relatively higher sensitivity (73.47 – 81.63%) as compared to the MRA (30.61 - 57.14%). However, the specificity of GPS was lower (34.69 – 73.47%) when compared with the MRA (98% by both criteria). The sensitivity increased with the severity of glaucoma for both MRA (52.17% for early cases and 60% for moderate glaucoma cases) and GPS (78.3% for early cases and 84% for moderate glaucoma cases).

The sensitivity and specificity values were extrapolated to calculate the likelihood ratios, to see the effect on the post-test probability of the disease. The MRA global classification tended to give a larger positive likelihood ratio (28.57 vs. 3.08), with a large effect on post-test probability, and the GPS global classification tended to give a better negative likelihood ratio (0.25 vs. 0.44) with a moderate effect on the post-test probability.

Complete agreement (classified in the same category by both the analysis) was observed in 36% of the normal and glaucoma patients [Tables 2 and 3]. Partial agreement (classified by one analysis as BL and by the other as either WNL or ONL) was observed in 38% of both the normal and glaucoma groups. There was a poor agreement between the overall MRA and GPS classifications; weighted *k* = 0.216 (95% CI: 0.119 – 0.315). GPS gave higher false positive results (28%) and MRA showed higher false negative results (42%).

**Table 2:** Distribution of glaucoma subjects among classifications

GPS* → MRA † ↓	WNL‡	BL§	ONL||	Total
WNL‡	2	6	13	21
BL§	0	1	13	14
ONL||	0	0	15	15
Total	2	7	41	

* GPS: Glaucoma probability score

† MRA: Moorfield’s regression analysis

‡ WNL: Within normal limits

§ BL: Borderline

|| ONL: Outside normal limits

**Table 3:** Distribution of healthy controls among classifications

GPS* → MRA † ↓	WNL‡	BL§	ONL||	Total
WNL‡	17	19	13	49
BL§	0	0	0	0
ONL||	0	0	1	1
Total	17	19	14	

* GPS: Glaucoma probability score

† MRA: Moorfield’s regression analysis

‡ WNL: Within normal limits

§ BL: Borderline

|| ONL: Outside normal limits

All the ONH stereometric parameters had a significant difference between the groups [Table 4]. The glaucoma patients had a larger disc area as compared to the healthy subjects (mean 2.43 mm^2^ vs. 2.24 mm^2^; *P* = 0.04). The parameters with the best discriminating ability between glaucoma and healthy subjects were cup-disc area ratio {AUROC(Area under ROC curve) = 0.842; Table 4}, rim-disc area ratio (AUROC = 0.842; Table 2), and rim area (AUROC = 0.832; Table 4). At a fixed specificity of 95%, the best sensitivity values were 57% for cup-disc area ratio and 55% for vertical cup-disc ratio. Table 5 shows a comparison of the GPS parameters between groups. The parameter with the best discriminating ability was the horizontal RNFL curvature (AUROC = 0.832).

**Table 4:** Comparison of stereometric parameters between groups

Parameter	Normal (n = 50)	Glaucoma (n = 50)	*P* value	AUROC*	Sensitivity at 95% Specificity (%)
	Mean	Mean			
Disc Area (mm^2^)	2.24 ± 0.41	2.43 ± 0.52	*P* = 0.04		
Cup Area (mm^2^)	0.61 ± 0.38	1.28 ± 0.65	*P* < 0.001	0.813	36.7
Rim Area (mm^2^)	1.63 ± 0.37	1.15 ± 0.41	*P* < 0.001	0.832	26
Cup/Disc Area Ratio	0.26 ± 0.15	0.51 ± 0.21	*P* < 0.001	0.842	57
Rim Area/Disc Area Ratio	0.74 ± 0.15	0.49 ± 0.21	*P* < 0.001	0.842	8
Cup Volume (mm^3^)	0.17 ± 0.18	0.48 ± 0.38	*P* < 0.001	0.792	35
Rim Volume (mm^3^)	0.43 ± 0.18	0.26 ± 0.15	*P* < 0.001	0.780	20
Mean Cup Depth (mm)	0.24 ± 0.11	0.36 ± 0.14	*P* < 0.001	0.760	24
Maximum Cup Depth (mm)	0.63 ± 0.22	0.79 ± 0.26	*P* < 0.001	0.682	23
Cup Shape Measure	-0.17 ± 0.08	-0.08 ± 0.08	*P* < 0.001	0.786	22
Cup/Disc Horizontal ratio	0.49 ± 0.21	0.70 ± 0.22	*P* < 0.001	0.775	41
Cup/Disc Vertical ratio	0.38 ± 0.23	0.63 ± 0.24	*P* < 0.001	0.823	55
FSM	1.27 ± 2,23	-1.23 ± 2.4	*P* < 0.001	0.805	14
RB	1.29 ± 0.75	0.17 ± 1.26_	*P* < 0.001	0.797	30

* AUROC: Area under receiver operating characteristic curve

**Table 5:** Comparison of glaucoma probability score parameters between groups

Parameter	Normals (n=50)	Glaucoma (n=50)	P value	AUROC*
	Mean	Mean		
Cup depth(mm)	0.64 ± 0.18	0.70 ± 0.19	*P* = 0.118	0.589
Horizontal RNFL† curvature	-0.03 ± 0.05	-0.09 ± 0.05	*P* < 0.001	0.832
Vertical RNFL† curvature	-0.11 ± 0.05	-0.16 ± 0.06	*P* < 0.001	0.753
Rim steepness	-0.19 ± 0.48	-0.52 ± 0.54	*P* < 0.001	0.702
Cup size	0.51 ± 0.20	0.70 ± 0.29	*P* < 0.001	0.734

* AUROC: Area Under receiver operating characteristic curve

† RNFL: Retinal nerve fibre layer

Table 6 shows the sectoral analysis of MRA and GPS classifications. The cut off point was chosen to be BL for MRA and ONL for GPS classifications. Overall, the GPS sectors had higher AUROCs than the MRA sectors. The sector with the best discriminating ability was nasal inferior (AUROC = 0.723) for MRA and temporal superior (AUROC = 0.860) for GPS. MRA sectors had better positive likelihood ratios (6.37-22.5), whereas, GPS sectors had better negative likelihood ratios (0.14 – 0.21).

**Table 6:** Sectoral analysis of Moorfield’s regression analysis and glaucoma probability score classifications

	AUROC*	Sensitivity (%)	Specificity (%)	+LR †	-LR†
Temp Superior					
MRA‡	0.682	39	98	19.5	0.622
GPS§	0.860	89	77	3.9	0.14
Temp Inferior					
MRA‡	0.716	47	96	11.75	0.55
GPS§	0.854	84	77	3.65	0.21
Nasal Superior					
MRA‡	0.712	45	98	22.5	0.56
GPS§	0.846	87	78	3.95	0.17
Nasal Inferior					
MRA‡	0.723	51	92	6.37	0.53
GPS§	0.847	84	76	3.5	0.21

* AUROC: Area under receiver operating characteristic curve

† LR: Likelihood ratio

‡ MRA: Moorfield’s regression analysis

§ GPS: Glaucoma probability score

The optic disc size was divided into small (< 1.87 mm ^2^), average sized (1.27 – 2.81 mm ^2^), and large sized (> 2.81 mm ^2^) depending on the normative database of a population with similar ethnicity.[6] The cutoff point was BL for MRA and ONL for GPS. The sensitivity of both MRA and GPS increased with the increase in disc size [Table 7]. Specificity for MRA did not vary much with the disc size, whereas, the same for GPS decreased markedly for larger sized discs.

**Table 7:** Effect of disc size on Moorfield’s regression analysis and glaucoma probability score classifications

	Small discs (<1.87) n = 13	Normal discs (1.87-2.81) n = 73	Large discs (>2.81) n = 14
MRA*			
Sensitivity	40	52.78	87.5
Specificity	100	97.3	100
GPS†			
Sensitivity	60	80.56	100
Specificity	100	75	20

* MRA: Moorfield’s regression analysis

† GPS: Glaucoma probability score Figures indicates in percentage

For GPS, the ‘false positive’ population had a larger disc area than the ‘true negative’ population (2.57 vs. 1.99 mm ^2^). Similarly for MRA, comparing the ‘false negative’ subjects with the ‘true positive’ ones, the disc area was smaller for the ones falsely labeled negative (2.28 vs. 2.64 mm ^2^).

## Discussion

The diagnosis of glaucoma in the early stages is a clinical challenge. Subjective evaluation of the ONH is difficult to reproduce because of a great inter-individual variability.[7] Established methods of objective ONH analysis such as the HRT have been utilized to diagnose and document ONH changes due to glaucoma. The purpose of this study was to compare the diagnostic performances of the operator-drawn, contour line dependent MRA and the contour line independent GPS, in patients with primary open angle glaucoma and normal healthy subjects.

The average age of the glaucoma patients was greater when compared with that of the controls. Previous studies by Zangwill *et al*,[8] Javier *et al*,[9] Harizman *et al*,[10] Coops *et al*,[11] and Burgansky-Eliash *et al*,[12] also reported similar results of healthy subjects being younger than the glaucoma patients. This might have been due to the fact that younger healthy subjects volunteered more often for participation in the study. The discriminatory value of parameters for normality could be exaggerated when using younger controls than in cases where it was due to age-related loss. In our study, the patients in the glaucoma group were slightly more myopic as compared to the controls; the refractive error was adjusted with proper focusing. The glaucoma patients who enrolled in our study had their IOP controlled either by medical or surgical means. Yet, the IOP measurements were higher (statistically significant) for glaucoma subjects. As expected, the MD and PSD of the patients in the moderate and early glaucoma group were significantly higher than those of the normal subjects.

The ONH parameters were evaluated and compared among the glaucoma and healthy subjects using HRT III. The average disc area of the normal subjects in our study was 2.24 mm ^2^. This was similar to the disc area of a mutually exclusive group of 275 normal eyes evaluated by HRT III in a previous study carried out in the center.[6] However, the disc area measured in the glaucoma group was larger (2.43 mm ^2^). One of the possible explanations for this could be a sampling bias in the training data. If early damage was difficult to detect in small optic discs by ophthalmoscopy, such discs were likely to be under represented among patients with glaucoma attending the centers. Similarly, if the damage was readily detectable in large discs, eyes with large discs could be relatively overrepresented. A more representative sample of glaucomatous optic disc damage could be drawn from subjects exhibiting glaucomatous visual field damage in a screening study, so that disc-related features did not influence the classifications.

The highest AUROCs among the HRT parameters were those of the rim area–disc area ratio and the cup area–disc area ratio followed closely by the rim area and vertical cup-disc ratios. Ferreras *et al*,[13] also reported results similar to the above-mentioned parameters. Both the linear discriminant functions in the HRT, Rb, and FSM had a similar distinguishing ability between the glaucoma and normal subjects (AUROC values 0.805 and 0.797, respectively). The GPS parameter with the highest AUROC was the horizontal RNFL curvature. The result was similar in various studies,[10–12] including the one by Swindale *et al*,[4] which showed that the same parameter had the highest distinguishing ability between the normal and glaucomatous subjects.

There was poor agreement between the overall MRA and GPS classifications. The agreement coefficient (*k*) was 0.216, which corresponded to a poor agreement. Burgansky-Eliash and associates[12] reported an agreement of 78.5% (*k*, 0.56) and Coops and associates[11] reported an agreement of 71% (*k*, 0.52). However, Javier *et al*,[9] reported a lower agreement in 56% of the cases (*k*, 0.34). The reason for this discrepancy in the current study and the study by Javier *et al*[9] might have been the number of eyes with early glaucoma (50% of our patients had early glaucoma). Various studies[8,12,14] reported that the sensitivity of both the algorithms decreases, especially for MRA,[10] in eyes with early glaucoma.

The MRA, developed to improve HRT diagnostic ability, considers variability in the area of the optic disc in the quantitative evaluation of the rim area. The MRA technique uses linear regression of the rim area by disc area to improve the HRT diagnostic capacity. In early glaucoma, a sensitivity of 59.6% and specificity of 72.3% have been reported.[10] Different sensitivities and specificities have been reported in the past for a number of reasons. First, the optic disc shapes and sizes vary widely in normal and open-angle glaucoma patients. Second, the sensitivities and specificities depend on the severity and stage of the glaucoma. Other studies have also shown the importance of disc size and race in the classification of glaucomatous and healthy eyes using the MRA. The new HRT III offers changes that attempt to increase diagnostic precision, including a larger normative database, and a new GPS algorithm. Until now, varied levels of diagnostic accuracy have been reported with the same.[8’^19^] In the current study, the MRA sensitivity ranges from 30.61 to 57.14%, which is inferior to that published in the studies previously.[8,10] The difference must have resulted from the fact that 50% of our patients had early glaucoma. The specificity is higher than that in previous studies.[8,10]

Regarding the GPS algorithm, we obtained a sensitivity slightly higher than in other studies, and the specificity was lower. Thus, the GPS had a tendency for higher sensitivity and lower specificity compared to the MRA. However, our best results were obtained when considering BL GPS values as negative results and BL MRA values as positive results. The positive likelihood ratios were higher for MRA, whereas, GPS gave better negative likelihood ratios. These results in the study population suggested that GPS provided better information for confirming a normal disc, whereas, MRA was most helpful in confirming a suspicion of glaucoma. These results were consistent with the studies done previously by Zangwill *et al*[8] and Ferreras *et al*.[13]

Both the MRA and GPS showed a lower sensitivity for smaller discs and higher sensitivity for larger discs. Although the specificity remained similar across varying disc sizes for MRA, the same decreased with increasing disc size for GPS. Also, previous studies[8,13] had shown a similar effect of disc size on the diagnostic accuracy of both the algorithms.

In conclusion, the overall agreement between the MRA and GPS classifications is poor. GPS has a better sensitivity and lower specificity than MRA. GPS and MRA show a decreased sensitivity for smaller discs and GPS shows a decreased specificity for larger discs.
